# The first insight into the genetic structure of the population of modern Serbia

**DOI:** 10.1038/s41598-021-93129-4

**Published:** 2021-07-07

**Authors:** Tamara Drljaca, Branka Zukic, Vladimir Kovacevic, Branislava Gemovic, Kristel Klaassen-Ljubicic, Vladimir Perovic, Mladen Lazarevic, Sonja Pavlovic, Nevena Veljkovic

**Affiliations:** 1 Vinca Institute of Nuclear Sciences, National Institute of the Republic of Serbia, University of Belgrade, Belgrade, Serbia; 2 Institute of Molecular Genetics and Genetic Engineering, University of Belgrade, Belgrade, Serbia; 3 Seven Bridges, Boston, MA USA; 4 Heliant Ltd, Belgrade, Serbia

**Keywords:** Genetics, Population genetics, Genetic variation

## Abstract

The complete understanding of the genomic contribution to complex traits, diseases, and response to treatments, as well as genomic medicine application to the well-being of all humans will be achieved through the global variome that encompasses fine-scale genetic diversity. Despite significant efforts in recent years, uneven representation still characterizes genomic resources and among the underrepresented European populations are the Western Balkans including the Serbian population. Our research addresses this gap and presents the first ever targeted sequencing dataset of variants in clinically relevant genes. By measuring population differentiation and applying the Principal Component and Admixture analysis we demonstrated that the Serbian population differs little from other European populations, yet we identified several novel and more frequent variants that appear as its unique genetic determinants. We explored thoroughly the functional impact of frequent variants and its correlation with the health burden of the population of Serbia based on a sample of 144 individuals*.* Our variants catalogue improves the understanding of genetics of modern Serbia, contributes to research on ancestry, and aids in improvements of well-being and health equity*.* In addition, this resource may also be applicable in neighboring regions and valuable in worldwide functional analyses of genetic variants in individuals of European descent.

## Introduction

In the era of high-throughput next generation sequencing (NGS) technology, the common goal for clinical use of sequencing data is the identification of pathogenic variants that can affect an individual’s health through linking genes and diseases^1–3^. The importance of elucidating fine-scale genetic diversity lies in understanding the genomic contribution to certain conditions, response to treatments, and in the application of acquired knowledge to clinical care and well-being of all people^4^. In the health context, equity will be achieved through the unbiased implementation of genomic medicine and evenly balanced structure of genomic analyses that comprise human specificities^5^. The misclassification of variants coming from data that do not include diverse subpopulations can potentially lead to misinterpretation of causative factors of disease and inadequate treatments of individuals from underrepresented segments^6^. Although large-scale variome studies, such as the 1000 Genomes Project (1kGP)^7^, Exome Aggregation Consortium (ExAC)^8^ and Genome Aggregation Database (gnomAD)^9^ have widely expanded our horizons on human diversity, numerous studies^10–13^ are demonstrating that there are many more population-specific variations than have been captured through these initiatives. In recent years, significant effort has been invested in addressing gaps in the composition of the global genomic landscape^6^.

Many countries have performed national studies to create population-specific variant panels and supplement the universal reference genome. These studies have a common aim in understanding genetic variability at the population level, as well as understanding and interpreting pathogenic variants and prioritizing candidate disease-causing genetic variation. The UK Biobank^14,15^ is a contemporary population genetics project with considerable sample sizes, set up to potentiate genetic and non-genetic determinants of the disease.

Other European projects aimed at applying a newly acquired dataset as a reference for clinical and medical sequencing projects are the Genome of the Netherlands^16^ and the Danish Reference Genome Project^17^. SweGen^18^ and Iceland's project^19^ are examples of national projects intended to assess genetic variability at a more detailed level in order to establish a control dataset for the local population.

Although European population genetics has been largely studied and well described, the Western Balkans including Serbia are underrepresented in the majority of cohorts. Modern Serbia is a landlocked Western Balkan country in south eastern Europe with a population of 7 million citizens (without Kosovo and Metohija)^20^. The population of Serbia comprises predominantly Serbs (83%), while there are also numerous minorities: Hungarians, Bosnians, Slovaks, Croatians, Albanians, Romanians, Bulgarians, and Macedonians. The population of Serbia is demographically old^21^ with a health profile predominantly burdened with cardiovascular diseases and cancer^22^. However, thus far, there have been no reports on the contemporary Serbian variome. Most of the research on Balkan populations was conducted on uniparentally inherited markers such as mitochondrial DNA (mtDNA) and the Y chromosome, focusing on population descent and haplogroup diversity^23–25^. One recent exception is a report that describes the sequencing and analysis of a genome from a contemporary individual of Serbian origin and which introduces tens of thousands of previously unknown variants^26^.

In this work, our focus was on common variants in the Serbian population sample and their functional impact. We created a catalogue of variants called after the clinical exome sequencing of 147 individuals from Serbia. Not only novel variants, but also variants that are frequent in the Serbian sample but much less frequent in the European population are identified as unique genetic characteristics of the studied population.

## Results and discussion

### Description and functional prediction of variants in the Serbian population sample

**Figure 1:**
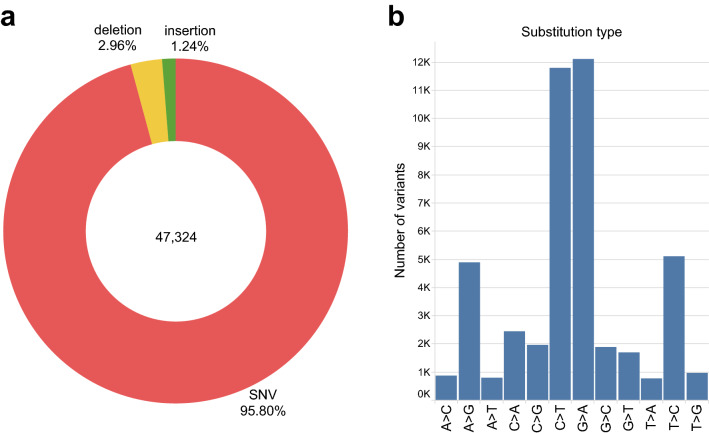
Serbian population sample variant classes and types. **(a)** Variant distribution by class shows that the vast majority of the total number of variants (47,324) are SNVs, followed by a significantly lower percent of deletions and insertions (**b)** SNV substitution type distribution.

After variant calling from a dataset that included sequencing data of 147 individuals, followed by sample and variant quality control (QC) filtration, we obtained a final multisample set reduced to 144 data samples. Average coverage was calculated per site for each BAM file and indicates appropriate quality of sequencing data (Supplementary Fig. S1). The final set has 47,324 elements, of which single nucleotide variants (SNVs) represent 95.80% (Fig. 1a). Indels together make 4.2% out of the overall dataset and indel distribution shape was shown in Supplementary Fig. S2. The transition-transversion ratio (Ti/Tv), as the measure of the overall SNV quality, was evaluated before and after QC and substitution type distribution as shown in (Fig. 1b). The Ti/Tv ratio increased from 2.92 to 2.97 which is in accordance with the quality expected for the exome data.

**Figure 2:**
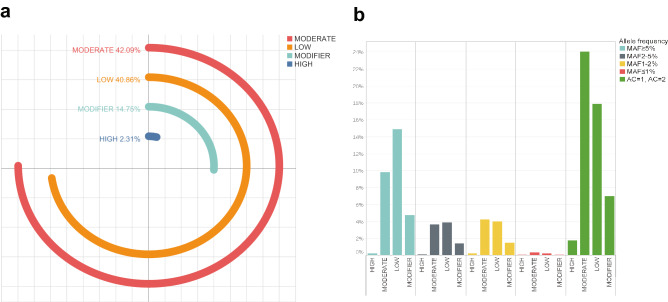
Functional effects of variants in the Serbian population sample **(a).** The distribution of variants across predicted categories. Modifiers and high functional impact variants are predicted for 17.06% of variants. The vast majority of variants are predicted to have moderate and low functional impact. (**b)** MAF categories are divided into functional impact subcategories. The value on the y-axis represents the number of variants. For each MAF category there are functional impact subcategories: high, moderate, low, and modifier.

Using the Variant Effect Predictor (VEP)^27^, the variants are assigned with effects using a rule-based approach to predict the effects that each allele of the variant may have on each transcript, according to Ensembl (https://m.ensembl.org/info/genome/variation/prediction/predicted_data.html). The impact rating is based on classification of the severity of functional effects. Functional classification showed that variants with moderate impact are the most abundant (19,918), whereas high impact variants make up 2.31% (1093) (Fig. 2, Supplementary Table S1). In our dataset 11.94% of the total number of variants was rare with minor allele frequency (MAF) of ≤ 1%, while the number of common variants (MAF ≥ 5%) on which we focused our research was 13,362 (28.24%). We calculated the number of singletons (Minor Allele Count, MAC = 1) and private doubletons (MAC = 2) separately and singletons make up 39.26% of the total number of variants, while private doubletons make up 1.03%. The cause of the high percent of singletons and private doubletons may be attributed to the small overall sample size^28^.

**Figure 3:**
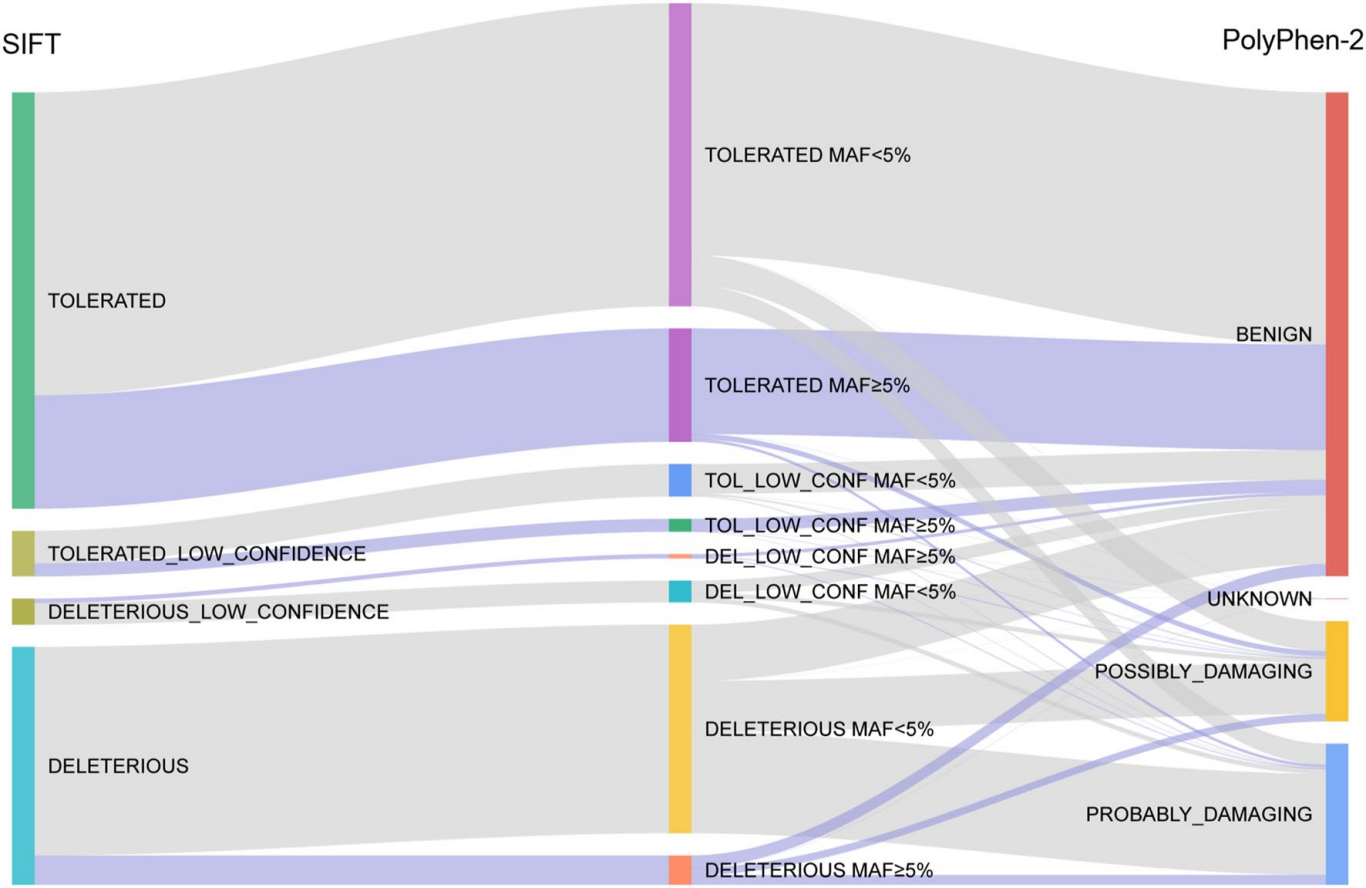
SIFT and PolyPhen-2 functional impact and different predictions. The figure presents the flow between the SIFT tool^29^ (left side of the chart) and PolyPhen-2 tool^30^ (right side of the chart) predictions. SNVs are differentiated between common variants, MAF ≥ 5% (grey lines) and rare variants, MAF < 5% (violet lines).

Functional annotation using the Ensembl VEP^27^ included embedded pathogenicity predictions with SIFT v5.2.2^29^ and PolyPhen-2 v2.2.2^30^. Pathogenicity prediction using SIFT was obtained for 40.33% (19,089) of the total number of SNVs and for 40.53% (19,183) using the PolyPhen-2 tool (Supplementary Fig. S3). The majority of variants are classified in similar categories by both tools (Fig. 3), i.e. variants predicted to be tolerated by SIFT were also seen as benign by PolyPhen-2 (49.29%), whereas most of those predicted to be deleterious by SIFT are perceived as damaging or probably damaging by PolyPhen-2. However, 9.42% of variants are cross-classified as deleterious and benign, while 3.25% variants are both tolerated and probably damaging (Supplementary Table S2). This SNVs set might be of particular interest because it is possible that their functional effects are subtle and thus, their significance may remain unexplored. In our future research we should explore these variants, particularly the mixed classified common ones (MAF ≥ 5%).

### The structure of the population of modern Serbia

The Balkan Peninsula, encompassing the larger part of Serbian territory, is situated at the crossroads of Central and Southeast Europe and was one of key areas in major migratory events that occurred after the Last Glacial Maximum^31–33^. The area of present-day Serbia has been inhabited since the Paleolithic Age^34^. As for the Neolithic Age, analysis of genome-wide DNA polymorphisms of populations bordering the Mediterranean coast, including Serbia, confirmed the hypothesis that the maritime coastal route was mainly used for the migration of Neolithic farmers to Europe^35^. The territory of modern-day Serbia faced Slavic migrations to Southeastern Europe in the sixth century, establishing several regional states recognized as tributaries to the Byzantine and Hungarian kingdoms. The Ottomans annexed the whole of modern-day Serbia by the mid-16th till the beginning of nineteenth century, and had a strong influence on the Serbian people, especially to the south of the country. From the end of the seventeenth century, the Habsburg Empire curtailed the Ottoman rule, expanding towards Central Serbia, but strongly influencing the north of Serbia—the Vojvodina province.

Here, we investigated the ancestry pattern and the genetic differentiation of the population of Serbia and the European populations of 1kGP. The Weir and Cockerham^36^ pairwise F_ST_ estimator showed, according to Wright’s classification^37^, small genetic distance between the population of Serbia and the European populations (TSI F_ST_ = 0.00342, CEU F_ST_ = 0.00343, IBS F_ST_ = 0.00344, GBR F_ST_ = 0.00415, FIN F_ST_ = 0.009), with a mean F_ST_ = 0.003 for the genetic difference between the total European population of the 1000 Genomes Project.

**Figure 4:**
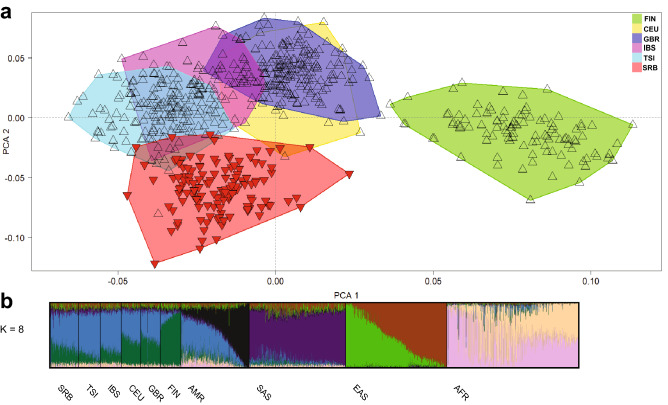
(**a**) Principal component analysis (PCA) of the Serbian population sample. The PCA of the study data combined with The 1000 Genomes Project Phase 3 data of European populations: *SRB* Serbian, *TSI*  Toscani in Italia, *IBS*  Iberian population in Spain, *CEU*  Utah Residents with Northern and Western European Ancestry, *GBR*  British in England and Scotland, *FIN*  Finnish in Finland. The figure represents the overlap of convex hulls of the population of Serbia with European populations of 1kGP, without the four outliers found in the population of Serbia while performing PCA. (**b**) Population structure analysis by applying the ADMIXTURE^38^. Distribution of the ancestry components for K = 8. Each bar plot represents the average ancestry proportions across individuals from the Serbian (SRB) and populations from 1kGP. Population codes: *SRB*  Serbian, *TSI*  Toscani in Italia, *IBS*  Iberian population in Spain, *CEU* Utah Residents with Northern and Western European Ancestry, *GBR*  British in England and Scotland, *FIN*  Finnish in Finland, *AMR *American Admixed, *SAS *South Asian, *EAS*  East Asian, *AFR*  African.

Next, we explored the overlap between the convex hulls of the population of Serbia and the European populations of 1kGP, and revealed that the Serbian population dataset mostly does not overlap with other European populations used for comparison (Central Europeans = CEU, Toscans = TSI, Iberians = IBS, British = GBR and Finnish = FIN) (Fig. 4a). We noticed that 7 Serbian samples overlap with the TSI population, while 2 samples overlap with the IBS and 2 with the CEU population. Thus, our findings strongly support the conclusion that Serbia needs its own population dataset. Note that four outliers were removed, though they are shown in Supplementary Figure S4.

Furthermore, we investigated the structure of the population of Serbia using the ADMIXTURE^38^ with a combined study sample and 1kGP dataset that contains all available populations i.e. Americans, South Asians, East Asians, Africans and Europeans including Toscani in Italia, Iberian population in Spain, Utah Residents with Northern and Western European Ancestry, British in England and Scotland and Finnish in Finland. With a model of 8 hypothetical ancestral components (K = 8) which was the lowest cross validation value (Supplementary Fig. S6), we confirmed that the population of Serbia shares ancestry components with the European populations (Fig. 4.b, Supplementary Fig. S5).

### Distribution of novel variants in the Serbian population sample

**Figure 5:**
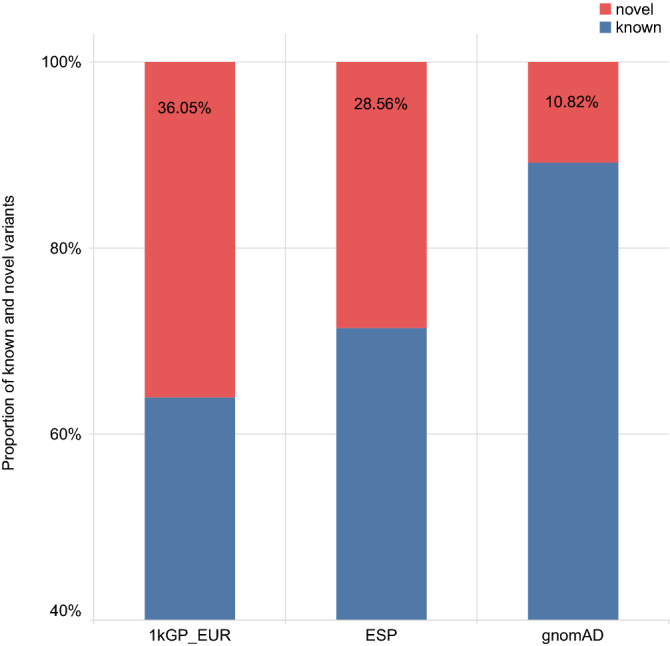
Ratio of known and novel variants in the Serbian population sample. Per reference database: 1kGP_EUR, gnomAD and NHLBI ESP.

Our variants dataset was intersected with reference databases 1kGP Phase 3, European population^7^, gnomAD v3.0^9^, and NHLBI ESP (https://evs.gs.washington.edu/EVS/)^39^ and revealed that 4972 (10.5%) variants are not present in any of them. These variants are referred to as novel and their presence in different databases is shown in Fig. 5. As expected, our dataset overlaps the most with gnomAD’s dataset which is the largest and which was mapped to the hg38 reference genome.

**Figure 6:**
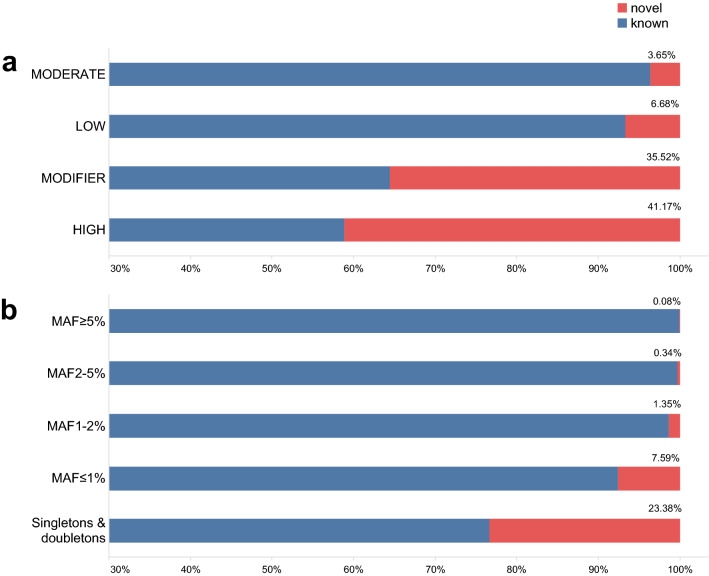
Novel variants in the Serbian population sample classified by predicted functional impact. **(a)** Percent of novel variants by functional impact relative to known variants found in all databases after intersection. (**b)** Percent of novel variants distributed across allele frequency categories.

Analyses of functional effects of novel variants revealed that the majority of novel variants (450) are in the category of high impact variants, followed by the modifier category. Furthermore, considering allele frequency and allele count, the majority of novel variants are rare in the Serbian population sample (Fig. 6). Note that according to Subramanian^40^ the resolution in identifying low frequency variants increases with the increase in the sample size, so we could expect an increase in novel variants with an increase in sample size of Serbian population.

Another interesting confirmation that Serbia needs its own dataset came from the experiment in which we intersect our sample with Europeans samples from HapMap 3 project^41^ data. After lifting HapMap dataset to hg38 reference genome we found only 8587 common variants out of 1,398,396 in CEU HapMap population.

### Missense variants frequent in the Serbian population sample: case studies

Using the CNVkit 0.9.1 toolkit^42^ we were able to determine the sex distribution in the Serbian sample. After the QC filtration, we excluded three samples due to the Het/Hom deviation and excess homozygosity. These three samples were sequenced together, thus we assume that the excess homozygosity is due to a sequencing error. Hence, not including these three samples prevented the final dataset bias to homozygous variants. After filtration we kept 61 female sample out of 62, and 83 male samples out of 85*.* Furthermore, we analysed the distribution of common variants found in Serbian sample in the remaining female and male samples (Table 1, Supplementary Table S3). As the literature search of overrepresented variants revealed that they were almost not investigated at all, we explored annotations of genes that harbour statistically significant variants (Supplementary Table S3) in the Gene Ontology database in order to better understand the processes and pathways that might be affected. In these analyses we were restricted to the sub-ontology biological processes (GO-BPO) and found that one gene is involved in the immune response and two genes participate in chemical synaptic transmission (Supplementary Table S5). Two genes, RHPN2 and BTNL2, do not have annotations in GO-BPO*.* One interesting coincidence is that the protein product affected by the variant that distinguishes the most studied population from other Europeans has the same name as an individual from the largest ethnic group in Serbia, the Serbs. The PSPH protein product SERB is a phosphoserine phosphatase and a member of the haloacid dehalogenase superfamily of hydrolytic dehalogenases^43^.

**Table 1:** Top 5 variants with the highest fold increase and statistical significance in the Serbian population sample compared to Europeans and their sex representation.

Gene	Variant	Substitution type	% of variants in the female sample	% of variants in the male sample	Fold increase	P-value
PSPH	chr7:g.56019730G > A	missense	36%	30%	163	1.9E-12
KIR2DL1	chr19:g.54773524A > G	missense	15%	11%	33	1.0E-19
KIR2DL1	chr19:g.54775225A > G	missense	21%	13%	21.25	1.0E-03
KIR2DL1	chr19:g.54775226 T > C	synonymous	21%	13%	21.25	9.8E-04
HLA-DRB5	chr6:g.32519465G > A	missense	20%	22%	5.10	5.2E-17

Statistical significance was determined by the one-tailed Fisher’s exact test with total frequencies from all 1kGP populations and after the Bonferroni correction the P-value threshold was P < 0.002.

Missense variants in these genes were further analyzed by using MutPred2^44^, a tool that predicts not only the pathogenicity as PolyPhen-2^30^ and SIFT^29^ but also the molecular mechanisms underlying the effects of variants predicted to be pathogenic (Table 2, Supplementary Table S4).

**Table 2:** Variants detected as frequent in the Serbian population sample that MutPred2^44^ predicted as affecting protein function.

Gene	Variant	AAS	MutPred2—molecular mechanisms
PSPH	rs79451216	R49W	Loss of relative solvent accessibility Loss of ADP-ribosylation at R49 Altered metal binding
		R49G	Loss of relative solvent accessibility Loss of helix Gain of ADP-ribosylation at R50 Altered metal binding, gain of methylation at R50
KIR2DL1	rs79002558	S88R	Loss of relative solvent accessibility Altered trans-membrane protein Altered ordered interface Gain of allosteric site at R89 Gain of ADP-ribosylation at S88 Altered DNA binding Altered metal binding Loss of N-linked glycosylation at N84
BTNL2	rs28362679	S334L	Altered transmembrane protein Loss of B-factor
		S334W	
HLA-DQB1	rs41552812	D89N	Altered ordered Gain of relative solvent accessibility

In the subsequent paragraphs we review a few genes that harbor variants characteristic of the population of Serbia.

The PSPH (Phosphoserine Phosphatase) gene codes for a member of the SerB protein family, a phosphoserine phosphatase involved in the biosynthesis of serine^45^. A recent study by Jia et al.^46^ showed that the PSPH loci is associated with the glycine level, while Byers et al.^47^ reported a decreased glycine level in a patient with PSPH mutations (V44G and G141S). A variant rs79451216 in PSPH, identified as frequent in the Serbian population sample, encompasses two alleles leading to amino acid substitution (AAS) of arginine at position 49 in the protein sequence, R49W and R49G. Of note, this variant is present in 22 out of 61 female samples and in 25 out of 83 male samples. However, sex differences for this and other variants have to be further investigated. MutPred2 showed that these substitutions affect the PSPH protein functions, while molecular mechanisms underlying this disturbance were predicted to be associated with phosphorylation and cleavage of the PSPH protein (Table 2). Thus far, there is no information about the effect of these variants at the level of metabolites affected by PSPH, but their proximity to the already described glycine decreasing variant^47^ can lead to the assumption of the same effect. Since glycine was shown to have antihypertensive and atheroprotective properties, as well as, to reduce risk of acute myocardial infarction^48,49^, gene variants lowering the glycine level in blood might increase susceptibility to various cardiovascular diseases. Ischemic heart disease and cerebrovascular diseases are the most dominant causes of death in Serbia^21,22^, and the rate of ischemic heart disease in Serbia was higher in comparison with all other European regions^22^. Although this can be attributed to several factors, our results for the first time implicate variations in the PSPH gene as a possible contributor to the high incidence of cardiovascular diseases in Serbia.

The KIR2DL1 (Killer cell immunoglobulin-like receptor 2DL1) codes for one of the receptors on natural killer cells, inhibiting their activity. KIR genes are located in one of the most variable regions of the human genome. Variability of KIR genes is represented by both the presence/absence of genes and sequence polymorphism, leading to high diversity among individuals, as well as different populations^50^. Ligands for this protein are the Human Leucocyte Antigen C (HLA-C) molecules and this interaction is involved in the immune response and dealing with various pathogen infections, including the Hepatitis C Virus (HCV)^51^. Variants in both KIR and HLA-C genes, as well as interleukin 28B (IL28B), affect the course of the HCV infection and the response to therapy^52,53^. Genotyping of these genes is therefore highly recommended in the clinical circumstances involving decisions about the anti-HCV treatment^52^. Since a variant in KIR2DL1 was found to have a 33-fold increase in frequency in the Serbian population sample compared to European populations of 1kGP it should be taken into account when genotyping HCV infected patients in Serbia for this gene. Additionally, this gene variant leads to an amino acid change that was shown in the MutPred2 analysis to be of functional importance (Table 2). Therefore, further consequences of this change for the interaction of KIR2DL1 and HLA-C, as well as additional effects on the immune response in the Serbian population should be tested. Of note, this variant was found in 9 out of 61 female samples and in 9 out of 83 male samples.

Finally, we identified two overrepresented variants in BTNL2 and HLA-DQB1 genes that are according to the literature linked to sarcoidosis, a multiorgan granulomatous inflammatory disease primarily affecting the lungs, but also lymph nodes, liver, spleen, skin, eyes, muscles, brain, kidneys and heart^54^. The BTNL2 (Butyrophilin-like 2) gene codes for a member of the immunoglobulin superfamily, which acts as an inhibitor of T cell activation^55^. BTNL2 is a susceptibility and progression factor for pulmonary sarcoidosis^56^ and polymorphisms in this gene are associated with phenotype expression of sarcoidosis^56^. Similarly, an HLA-DQB1 haplotype is strongly associated with severe sarcoidosis^57^. MutPred2 analysis showed that two variants in the BTNL2 and HLA-DQB1 genes, leading to three AAS – S334L and S334W in BTNL2 and D89N, affect the functions of these proteins (Table 2). Pulmonary sarcoidosis in Serbian patients is most often followed by ocular sarcoidosis, as the first most common site of extrapulmonary sarcoid manifestations^58^. Some clinical features of these patients differ from those in other European populations, with more common neuro-ophthalmologic lesions^58^. This difference could be associated with variants in BTNL2 and HLA-DQB1, which we found to be frequent in the Serbian population sample and functionally important, and should be further investigated. The HLA-DQB1 rs41552812 variant was found in 13 out of 61 female samples and in 19 out of 83 male samples. There is an additional example of specificity of the Serbian genomic profile related to sarcoidosis^59^.

## Conclusion

Large-scale variome studies have significantly increased our understanding of the diversity in the human population, however, its composition is still broadly biased towards some populations.

In this study we aimed to address the gap in the European genomic landscape and to the best of our knowledge provided the first ever dataset of variants in the population of contemporary Serbia. Variants are described in detail according to allele frequency, presence in key population databases and functional impact as interpreted by several state-of-the-art tools. Ancestry analyses demonstrated that the Serbian population differs relatively little from other European populations, yet we discerned and reported some unique genetic characteristics as several variants that are novel or significantly overrepresented in the Serbian population sample. These insights will be further evaluated and broadened in larger genomic studies linking genes and diseases. Nevertheless, the variant’s catalogue obtained contributes to our understanding of the genetics of modern Serbia and more adequate functional interpretation in the context of precision medicine and health equity.

## Methods

### Study population

The study cohort consists of 147 individuals unrelated either by consanguinity or affinity. All participants of the study are of Serbian descent, declared as Serbian native speakers. The distribution of the participants by geographical regions was as follows: 35% Belgrade, 32% Vojvodina, 15% central-west region of Serbia, 15% southern Serbia, and 3% eastern region of Serbia. The distribution roughly reflects the overall population density of the country, being the most populated in Belgrade and Vojvodina, following the west and south of Serbia and the lowest in the eastern region of Serbia.

This study was approved by the Ethics Committee of the Institute of Molecular Genetics and Genetic Engineering, University of Belgrade, in accordance with the guidelines of the 1975 Declaration of Helsinki (6th revision, 2008). Informed consent was obtained from all participants included in this study.

### DNA sequencing

Total genomic DNA was isolated from peripheral blood using the QIAamp DNA Blood Mini Kit (Qiagen, Hilden, Germany). Sequencing was performed in a time span from 2014 to 2020. The sequencing technology was MiSeq Illumina using the TruSight-One Illumina (May 2014) sequencing panel for target exome sequencing, which consists of 4813 disease-associated genes (62,000 exons and nearby exon–intron boundaries) (12 Mb genome). Multiplex using 125 395 probes designed according to the NCBI37/hg19 reference genome—all functionally tested.

### Variant calling

Germline SNP and Indel variant calling was performed following the Genome Analysis Toolkit (GATK, v4.1.0.0) best practice recommendations^60^. Raw reads were mapped on the UCSC human reference genome hg38 using a Burrows-Wheeler Aligner (BWA-MEM, v0.7.17)^61^. Optical and PCR duplicate marking and sorting was done using Picard (v4.1.0.0) (https://broadinstitute.github.io/picard/). Base quality score recalibration was done with the GATK BaseRecalibrator resulting in a final BAM file for each sample. The reference files used for base quality score recalibration were dbSNP138, Mills and 1000 genome gold standard indels and 1000 genome phase 1, provided from the GATK Resource Bundle (last modified 8/22/16).

After data pre-processing, variant calling was done with the Haplotype Caller (v4.1.0.0)^62^ in the ERC GVCF mode to generate an intermediate gVCF file for each sample, which were then consolidated with the GenomicsDBImport (https://github.com/Intel-HLS/GenomicsDB) tool to produce a single file for joint calling. Joint calling was performed on the whole cohort of 147 samples using the GenotypeGVCF GATK4 to create a single multisample VCF file.

Considering that target exome sequencing data in this study does not support Variant Quality Score Recalibration, we selected hard filtering instead of VQSR. We applied hard filter thresholds recommended by GATK to increase the number of true positives and decrease the number of false positive variants. The applied filtering procedures following the standard GATK recommendations^63^ and metrics evaluated in the quality control protocol were for SNVs: FS, SOR, ReadPosRankSum, MQRankSum, QD, DP, MQ, and for indels: FS, SOR, ReadPosRankSum, MQRankSum, QD, DP.

Furthermore, on a reference sample (HG001, Genome In A Bottle) validation of the GATK variant calling pipeline was conducted and 96.9/99.4 recall/precision score was obtained. All steps were coordinated using the Cancer Genome Cloud Seven Bridges platform^64^.

### Quality control and annotation

To assess the quality of the obtained set of variants, we calculated per-sample metrics with Bcftools v1.9 (https://github.com/samtools/bcftools), such as the total number of variants, mean transition to transversion ratio (Ti/Tv) and average coverage per site with SAMtools v1.3^65^ calculated for each BAM file. We calculated the number of singletons and the ratio of heterozygous to non-reference homozygous sites (Het/Hom) in order to filter out low-quality samples. Samples with the Het/Hom ratio deviation were removed using PLINK v1.9 (www.cog-genomics.org/plink/1.9/)^66^. We marked the sites with depth (DP) < 20 and genotype quality (GQ) ≤  20 and excluded variants where more than 70% of genotypes did not pass the filters.

Deviation from the Hardy–Weinberg equilibrium (HWE) was calculated using VCFtools v0.1.13^67^ with a threshold for HWE of p < 0.0001 below which the variants were excluded. All variants failing the quality control (QC) steps were removed. All subsequent analyses were performed using clean post-QC datasets.

We used the Ensembl Variant Effect Predictor (VEP, ensembl-vep 90.5)^27^ for functional annotation of the final set of variants. Databases that were used within VEP were 1kGP Phase3, COSMIC v81, ClinVar 201706, NHLBI ESP V2-SSA137, HGMD-PUBLIC 20164, dbSNP150, GENCODE v27, gnomAD v2.1 and Regulatory Build. VEP provides scores and pathogenicity predictions with Sorting Intolerant From Tolerant v5.2.2 (SIFT)^29^ and PolyPhen-2 v2.2.2^30^ tools. For each transcript in the final dataset we obtained the coding consequences prediction and score according to SIFT and PolyPhen-2. A canonical transcript was assigned for each gene, according to VEP.

### Serbian sample sex structure

To determine the sex structure of the Serbian population sample we used the CNVkit 0.9.1 toolkit^42^. We evaluated the number of mapped reads on sex chromosomes of each sample BAM file using the CNVkit to generate target and antitarget BED files.

### Description of variants

In order to investigate allele frequency distribution in the Serbian population sample, we classified variants into four categories according to their minor allele frequency (MAF): MAF ≤ 1%, 1–2%, 2–5% and ≥ 5%. We separately classified singletons (AC = 1) and private doubletons (AC = 2), where a variant occurs only in one individual and in the homozygotic state.

We classified variants into four functional impact groups according to Ensembl (http://grch37.ensembl.org/info/genome/variation/prediction/predicted_data.html): HIGH (Loss of function) that includes splice donor variants, splice acceptor variants, stop gained, frameshift variants, stop lost and start lost. MODERATE that includes inframe insertion, inframe deletion, missense variants. LOW that includes splice region variants, synonymous variants, start and stop retained variants. MODIFIER that includes coding sequence variants, 5’UTR and 3’ UTR variants, non-coding transcript exon variants, intron variants, NMD transcript variants, non-coding transcript variants, upstream gene variants, downstream gene variants and intergenic variants.

In order to investigate the rate of overlap with reference databases and to determine novel variants, we compared our dataset with publicly available reference databases: gnomAD v3.0 genome (https://gnomad.broadinstitute.org/)^9^, NHLBI Exome Sequencing Project database vesp6500 (https://esp.gs.washington.edu/drupal/)^39^ and the European population from the 1kGP Phase 3 (https://www.internationalgenome.org/home)^7^. Populations included in the mentioned reference databases which we used to compare our dataset with, are from the following databases: (1) gnomAD: African/African-American, Amish, Latino/Admixed American, Ashkenazi Jew, East Asian, Finnish, Non-Finnish European, Middle Eastern, South Asian and Other, (2) NHLBI ESP: European American and African American, (3) 1kGP: European population.

The comparative datasets were cleaned up to fulfill the quality control standards. Both ours and comparative protocols included duplicate removal and recalibration during pre-processing, the use of hard filters and genotype and variant level filtering that included investigation of the Hardy–Weinberg equilibrium deviation, Het/Hom deviation, genotype missingness rate, GQ and DP quality control. Additional filtering methods were applied in order to respond to the specific needs of these comparative dataset analyzes, such as Support Vector Machine filtering (SVM), Variant Quality Score Recalibration (VQSR), identification and merging of candidate intervals and multi-sample clustering and genotyping^7,9,39^.

To compare our dataset with the previously mentioned databases, we used ANNOVAR v2019Oct24^68^ with a filter-based annotation option. Overlapping variants were identified comparing start and end positions, as well as the same observed alleles. Furthermore, we used BedTools v2.29.2^69^ to intersect multiple outputs from ANNOVAR to find variants that were not overlapping with any of the databases.

We conducted an intersection of our sample study data using PLINK-1.9, with HapMap 3 panel^41^ in order to investigate the overlap with data with a high degree of accuracy. HapMap dataset was previously lifted on hg38 reference genome with liftOver tool (https://genome.ucsc.edu/util.html).

### Population structure analyses

Principal Component Analysis (PCA) was used in order to estimate the ancestry of the Serbian population sample relative to other world populations. PCA was performed using the PLINK v1.9 (www.cog-genomics.org/plink/1.9/)^64^ software on the Serbian population sample and 1kGP Phase 3^7^ European populations dataset as a reference dataset. European populations included in 1kGP and considered in this examination are Toscani in Italia (TSI), Finnish in Finland (FIN), British in England and Scotland (GBR), Iberian Population in Spain (IBS) and Utah Residents with Northern and Western European Ancestry (CEU). In order to investigate the overlap with the European populations in more detail, we added convex hulls on the PCA plot.

To reduce the correlation between SNPs, study data was pruned for variants that are in linkage disequilibrium (LD). The LD-pruned dataset was generated using the PLINK v1.9 –indep-pairwise option, with parameters of the 50 kb window in which LD is calculated between each SNV pair and with the r^2^ > 1.5 threshold above which SNPs were removed. To ensure that only common (MAF ≥ 5%) variants are considered in the analysis, we set –maf to 0.05. The number of SNVs to shift the window at each step was set to 5. To reduce the size of the reference dataset to the size of the Serbian population sample, we filtered the reference dataset with the Serbian population sample SNVs. In order to compute the joint principal components of the reference and study population, the two datasets were merged and PCA was performed on the combined data.

Furthermore, the pairwise F_ST_ Weir and Cockerham^36^ estimator was calculated between the population of Serbia and the European populations of 1kGP: Toscani in Italia, Finnish in Finland, Iberian population in Spain, Utah Residents (CEPH) with Northern and Western European Ancestry, British in England and Scotland, using vcftools v0.1.13^67^.

We used ADMIXTURE v1.3.0^38^ to estimate study sample ancestries and further investigate the study sample structure. The 1000 Genome Project data was used in combination with our study sample in order to estimate individual ancestry. The five-fold cross-validation error was computed with K = 2 to 12 in order to obtain the optimal K value (Supplementary Fig. S6). The plots were visualized using the Pong software^70^.

### Overrepresented variants

To examine the variants that are significantly overrepresented in the population of Serbia compared to European populations of 1kGP, we calculated the fold increase in AF as AF_(Serbian)_/AF_(European)_ and singled out variants with a fold increase > 5. European populations included in 1kGP and considered in this examination are Toscani in Italia (TSI), Finnish in Finland (FIN), British in England and Scotland (GBR), Iberian Population in Spain (IBS) and Utah Residents with Northern and Western European Ancestry (CEU).

For the variants that are overrepresented in the population of Serbia (fold increase > 5 when compared with the European populations of 1kGP), we used the one-tailed Fisher’s exact test to measure significant differences in variant distributions between the population of Serbia and the populations of 1kGP. After the Bonferroni correction the P-value was considered significant at threshold P < 0.002.

### Gene ontology annotations

For identification of biological processes in which the 16 genes with frequent variants in the Serbian population are involved, the Biological Process Ontology (BPO) in Gene Ontology (GO) was used, release 2020-07-16. GO terms were filtered by Evidence Codes: EXP, IDA, IPI, IMP, IGI, IEP, TAS and IC.

### Functional analysis of missense variants

Missense variants frequent in the Serbian population were analysed using the MutPMissense variants frequent in the Serbian population were analysed using the MutPred2 web server^44^. MutPred2 is a machine-learning method for predicting pathogenicity of amino acid substitutions (AAS), which integrates genetic and molecular data. It provides a general pathogenicityred2 web server^44^. MutPred2 is a machine-learning method for predicting pathogenicity of amino acid substitutions (AAS), which integrates genetic and molecular data. It provides a general pathogenicity prediction, represented by the MutPred2 score, and a ranked list of specific molecular alterations potentially affecting the phenotype. MutPred2 estimates involvement of a missense variant in several structural and functional properties, including secondary structure, signal peptide and transmembrane topology, catalytic activity, macromolecular binding, post-translational modifications, metal-binding and allostery. We used the MutPred2 score ≥ 0.5 for predicting pathogenicity of a missense variant and p value ≤ 0.05 for predicting affected molecular mechanisms and motives.

Additional hypotheses about the functional effects of missense variants frequent in the Serbian population were created through a comprehensive manual search of available literature.

## Supplementary Information

Supplementary Information.

Supplementary Dataset 1.

Supplementary Dataset 2.

## Data Availability

The dataset generated and analysed during the current study is deposited and available as a single multisample VCF file at the European Variation Archive (EVA), https://www.ebi.ac.uk/ena/browser/view/ERA3199532.

## Code availability

We provided Common Workflow Language (CWL)^71^ code workflow that was obtained and used on the Seven Bridges Cancer Genome Cloud platform in order to perform variant calling according to GATK v4.1 best practice recommendations, as Supplementary information json format files Supplementary_file_1.json, Supplementary_file_2.json and Supplementary_file_3.json. Supplementary_file_1.json contains cwl workflow code that we used for data pre-processing and variant calling with Haplotype caller in ERC GVCF mode, Supplementary_file_2.json contains cwl workflow code for joint calling and Supplementary_file_3.json contains cwl workflow code that we used to perform hard filtering.
