# Supplementary material for: The first insight into the genetic structure of the population of modern Serbia
Source: Sci Rep. 2021 Jul 7;11:13995. doi: 10.1038/s41598-021-93129-4 (PMC8263702; doi:10.1038/s41598-021-93129-4)
Supplement: Supplementary file 1 — Supplementary Information. [file 41598_2021_93129_MOESM1_ESM.docx]

# Supplementary Data

**for**

The first insight into the genetic structure of

population of modern Serbia

Tamara Drljaca^1^, Branka Zukic^2^, Vladimir Kovacevic^3^, Branislava Gemovic^1^, Kristel

Klaassen-Ljubicic^2^, Mladen Lazarevic^3^, Vladimir Perovic^1^, Sonja Pavlovic*^2^ &

Nevena Veljkovic*^1,4^

^1^ Vinca Institute of Nuclear Sciences, National Institute of the Republic of Serbia,

University of Belgrade, Belgrade, Serbia

^2^ Institute of Molecular Genetics and Genetic Engineering, University of Belgrade,

Belgrade, Serbia

^3^ Seven Bridges, Boston, Massachusetts, USA

^4^ Heliant Ltd, Belgrade, Serbia

*Joint Corresponding authors

email: [nevenav@vin.bg.ac.rs,](mailto:nevena.veljkovic@vin.bg.ac.rs) [sonya@imgge.bg.ac.rs](mailto:sonya@imgge.bg.ac.rs)

## Average Coverage

Average coverage was calculated per position per sample, using SAMtools v.1.3. [1]

and the overall population sample average coverage is 88.25. Supplementary Figure S1

shows the average coverage of each sample after target exome sequencing that is

calculated per position and percent of coverage higher than the limit (20X) for each

sample. Considering that the vast majority of samples show a large percent of coverage

higher than limit, average coverage of samples indicates the appropriate quality of

sequencing data.


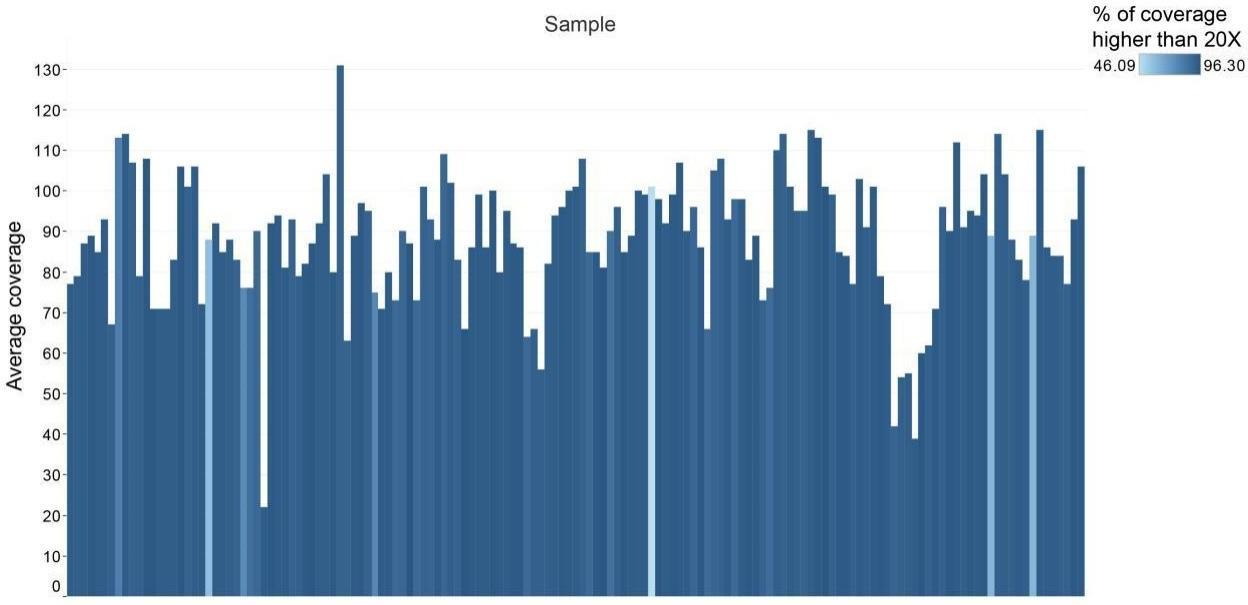


**Supplementary Figure S1** Target exome sequencing average coverage per sample. The y-axis

represents the average coverage calculated per position for each sample and the x-axis with

each bar represents individual samples. The color scale is used to represent percent of coverage

higher than the limit (20X) for each sample. The percent of coverage higher than the limit for

each sample in this study is in the range of 46.09 to 96.30.

## Indel Distribution

A total of 1987 indels was discovered in the Serbian population sample, out of which

697 were in lengths divisible with 3, ranging from 3 to 60 bp. We marked indels that

have lengths divisible by 3 as 3n indels and indels not divisible by 3 as non-3n indels,

where n is an integer [2]. We expect that 3n indels do not cause frameshift mutation.

Indel distribution shape (Supplementary Figure S2.) indicates an expected high number

of 1 bp indels, considering the high frequency of short indels [3] and the possibility of

PCR errors. According to Variant Effect Predictor (VEP) [4] annotation, in Serbian

population sample 552 indels were described with frameshift variant consequence.


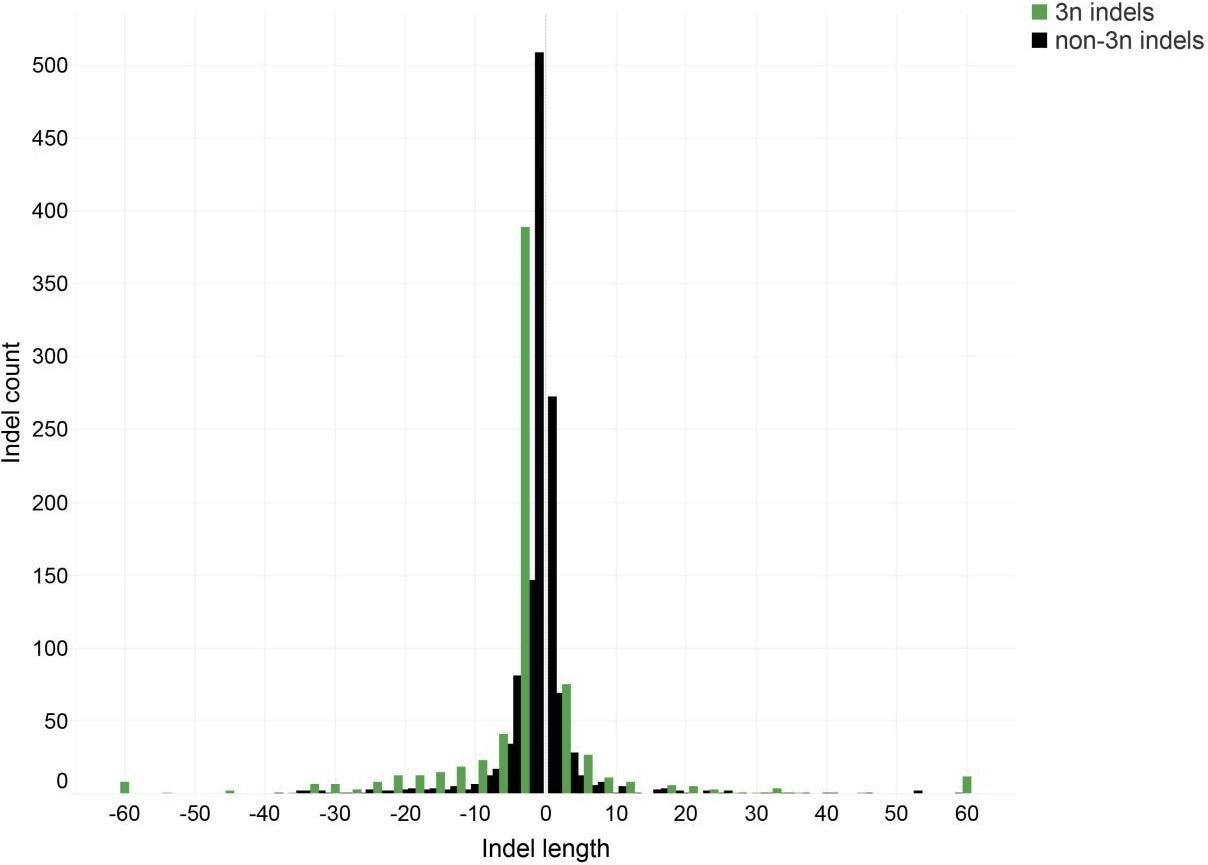


**Supplementary Figure S2.** Indel distribution. Indel length on the x-axis represents the length

of each indel, thus Indel length > 0 shows insertions and Indel length < 0 shows deletions. Indel

count on the y-axis shows the number of indels of a certain length. Shape of indel distribution

demonstrates a high number of 1 bp indels, as well as indels with lengths divisible with 3, which

are labeled with green as 3n indels. Indels not divisible by 3 are labeled as non-3n indels.

## Pathogenicity prediction of variants using SIFT and PolyPhen-2 tools

The distribution of variants by SIFT [5] and PolyPhen-2 [6] prediction categories is

shown in Supplementary Figure S3. SIFT tool predicts tolerated variants as most

represented (Supplementary Fig. S3a) with 57.36%, additionally PolyPhen-2 tool

supports this prediction with 66.67% benign variants (Supplementary Fig. S3b).


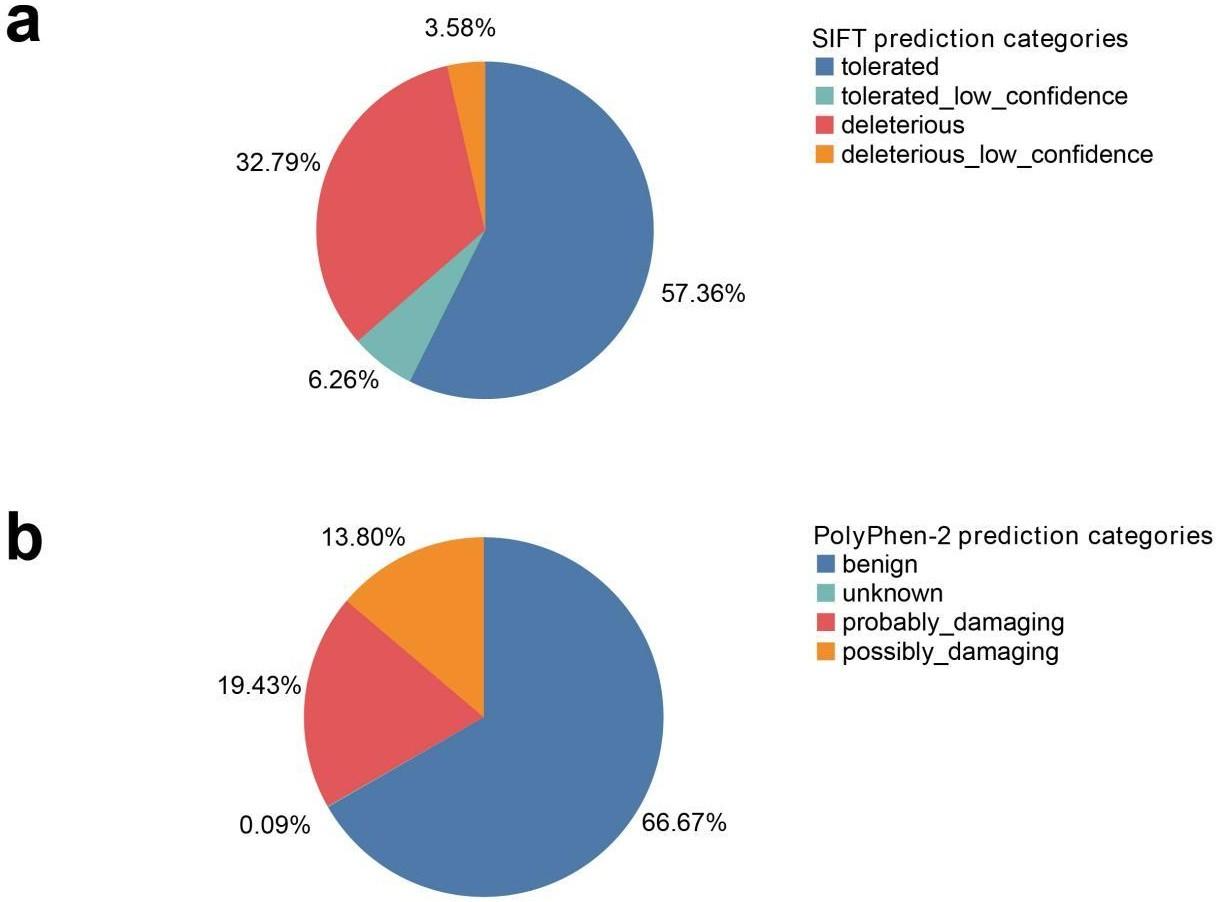


**Supplementary Figure S3** Distribution of variants by SIFT [5] and PolyPhen-2 [6]

pathogenicity prediction categories. **a**. Distribution of variants by SIFT tool pathogenicity

prediction categories with tolerated variants showed to be most abundant. **b.** Distribution of

variants by PolyPhen-2 tool prediction categories, with benign variants prevailing.

# Population structure analysis

**
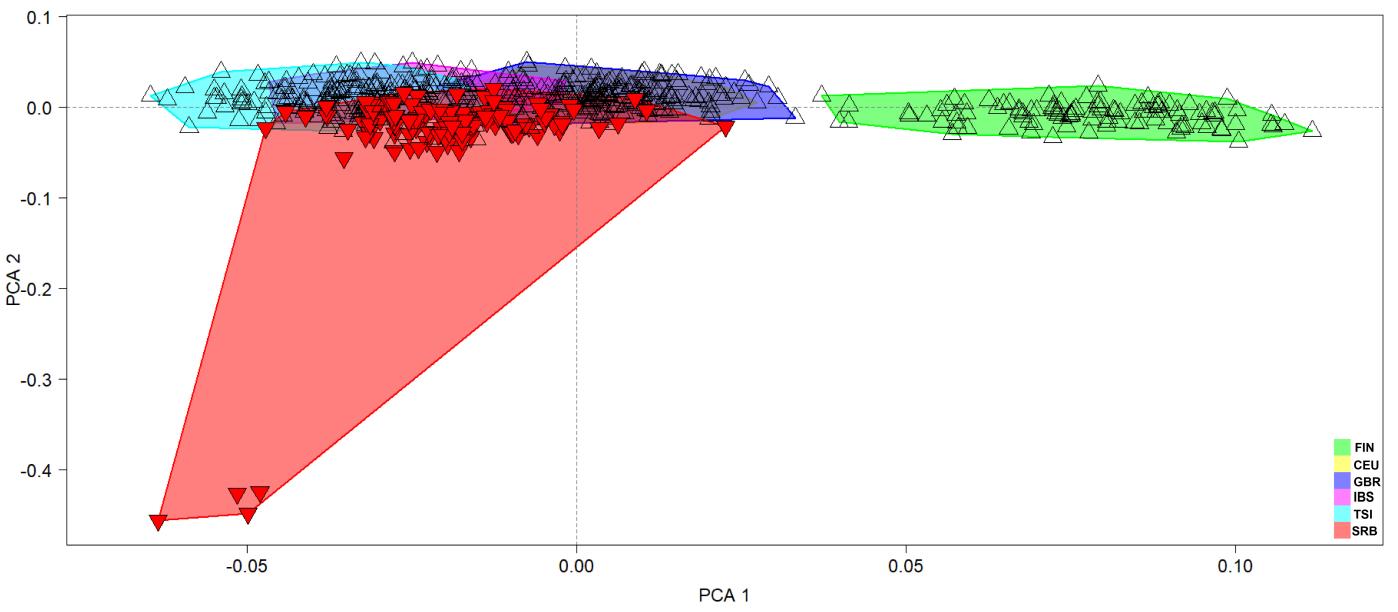
**

**Supplementary Figure S4 Principal Component Analysis with convex hulls.** The PCA of the population of Serbia combined with the European populations of 1kGP (TSI=Toscani in Italia, IBS=Iberian population in Spain, CEU=Utah Residents with Northern and Western European Ancestry, GBR=British in England and Scotland, FIN=Finnish in Finland) shows the four outliers in the population of Serbia (SRB).

We were particularly interested to investigate the loading values for the statistically significant variants found in the population of Serbia (Supplementary Table S3). The results showed that the only variant that was included in PCA (rs79002558) has relatively high negative loadings along both PC1 and PC2. We provided loading values in Supplementary_file_3.txt with loading values for the variants used in PCA.


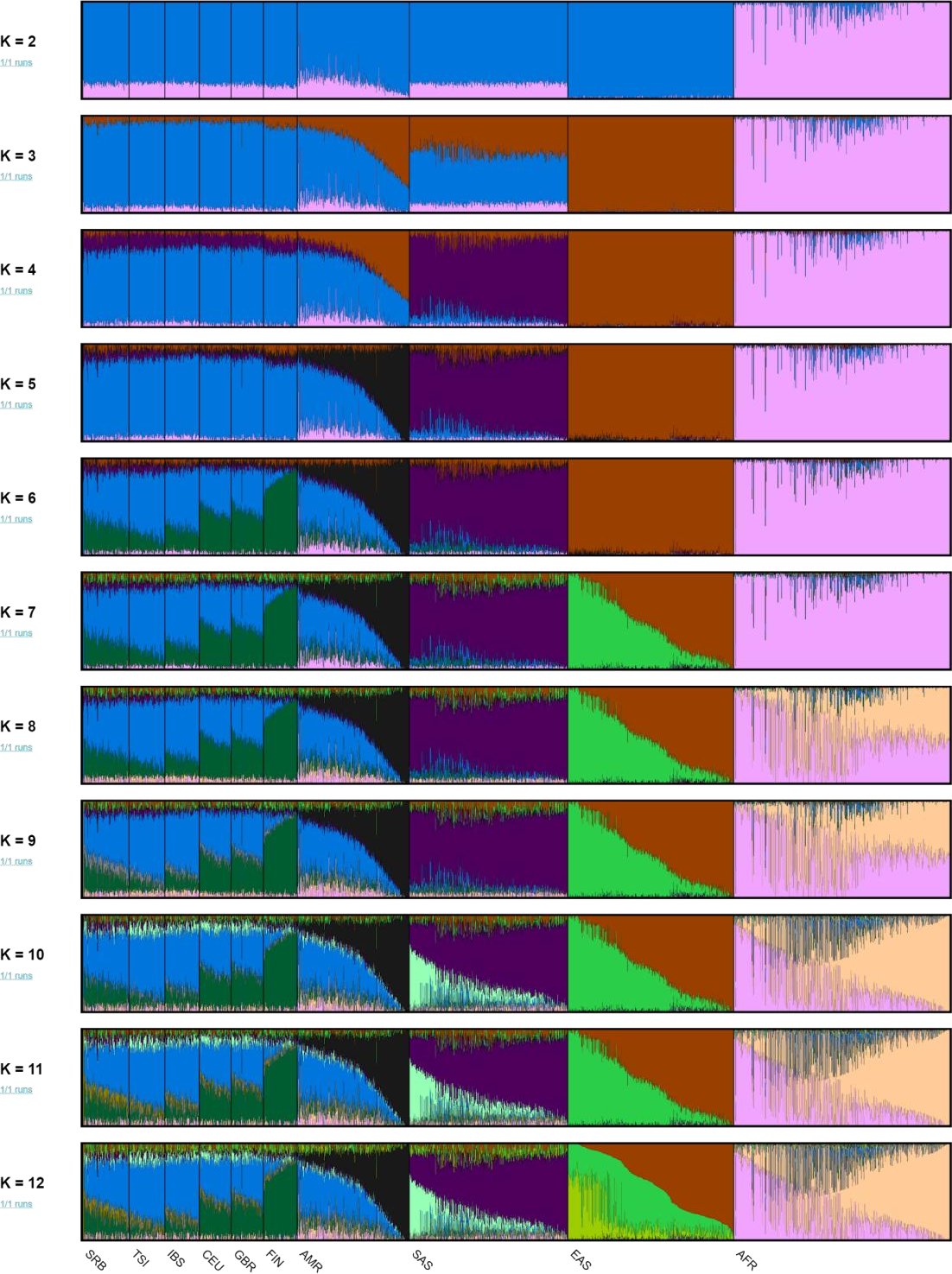


**Supplementary Figure S5** Admixture [7] results from K=2 to K=12. The population codes are:

SRB=Serbian, TSI=Toscani in Italia, IBS=Iberian population in Spain, CEU=Utah residents

(CEPH) with Northern and Western European Ancestry, GBR=British in England and Scotland,

FIN=Finnish in Finland, AMR=Ad Mixed American, SAS=South Asian, EAS=East Asian,

AFR=African


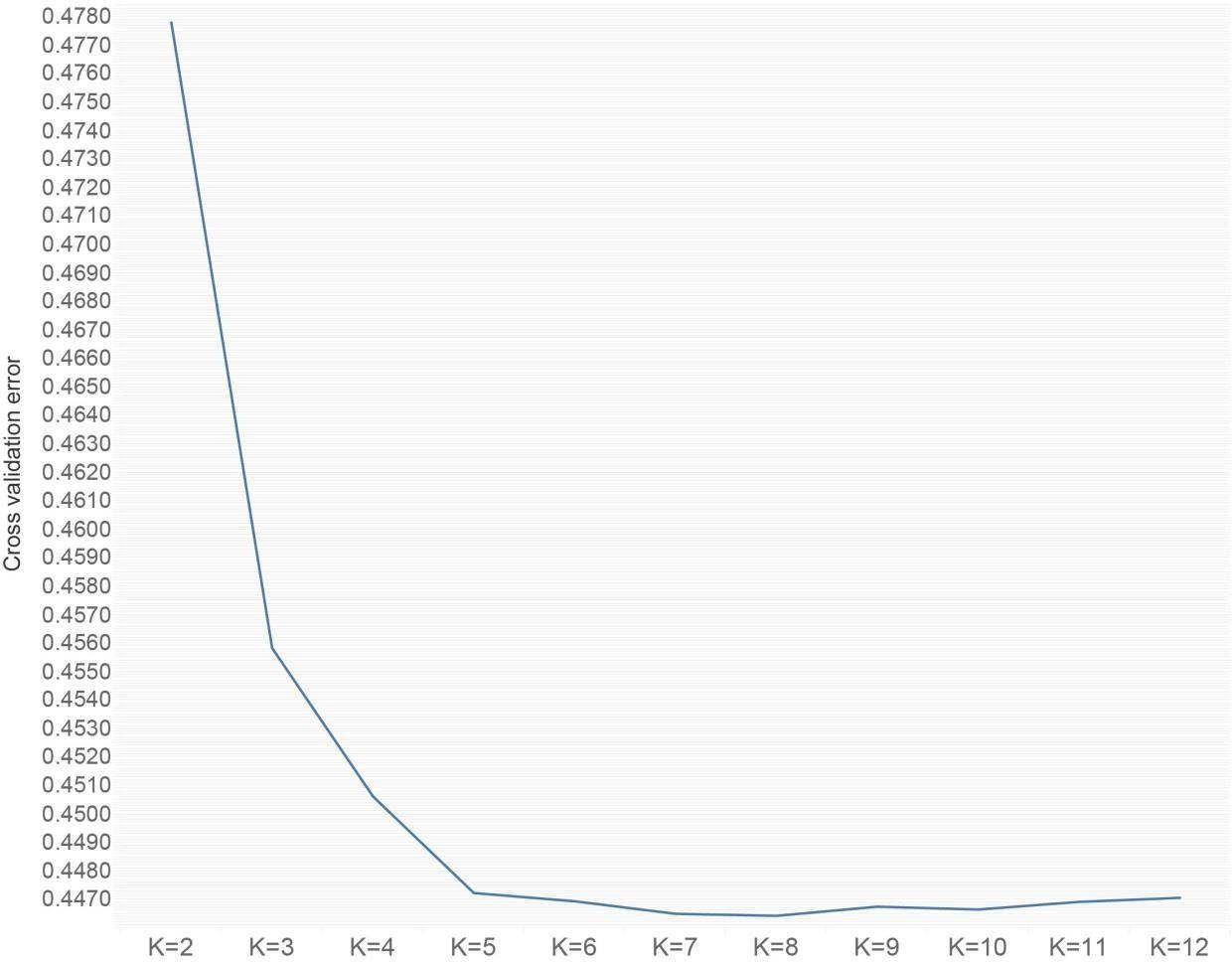


**Supplementary Figure S6.** Cross validation error at different K values, K=2 to K=12

# SUPPLEMENTARY TABLES

| **Functional impact** | | | | | |
| --- | --- | --- | --- | --- | --- |
|  | HIGH | MODERATE | LOW | MODIFIER | **Total MAF** |
| Singletons | 690  (1.46) | 8911  (18.83) | 6439  (13.60) | 2541  (5.37) | 18581  (39.26) |
| Doubletons | 27  (0.06) | 241  (0.51) | 127  (0.27) | 91  (0.20) | 486  (1.03) |
| MAF<≤1% | 131  (0.27) | 2553  (5.40) | 2201  (4.65) | 766  (1.62) | 5651  (11.94) |
| MAF1-2% | 89  (0.18) | 2031  (4.30) | 1922  (4.06) | 730  (1.54) | 4772  (10.08) |
| MAF2-5% | 57  (0.12) | 1783  (3.76) | 1932  (4.08) | 700  (1.47) | 4472  (9.45) |
| MAF>≥5% | 99  (0.21) | 4399  (9.30) | 6713  (14.18) | 2151  (4.54) | 13362  (28.24) |
| **Total Impact** | 1093  (2.31) | 19918  (42.09) | 19334  (40.86) | 6979  (14.75) | 47324  (100) |

**Supplementary Table S1** The number of variants (and percentage in brackets) found in

Serbian population sample, categorized by functional impact and Minor Allele frequency

(MAF).

| **PolyPhen-2** | benign | Probably_ damaging | Possibly_ damaging | unknown |
| --- | --- | --- | --- | --- |
| **SIFT** |  |  |  |  |
| tolerated | 9409  (49.29%) | 621  (3.25%) | 915  (4.79%) | 5  (0.03%) |
| Tolerated_  low_ confidence | 1107  (5.80%) | 32  (0.17%) | 49  (0.26%) | 7  (0.04%) |
| deleterious | 1799  (9.42%) | 2923  (15.31%) | 1537  (8.05%) | 1  (0.005%) |
| Deleterious_  low_ confidence | 410  (2.15%) | 138  (0.72%) | 134  (0.70%) | 2  (0.01%) |

**Supplementary Table S2** The number of variants that overlap between SIFT [5] and

PolyPhen-2 [6] functional prediction categories and percentage in brackets.

| **Gene** | **Variant** | **HGNC** | **Functional impact** | **Substitution type** | **% of variants in female sample** | **% of variants in male sample** | **Allele frequency** | | **Fold increase** | **P-value** |
| --- | --- | --- | --- | --- | --- | --- | --- | --- | --- | --- |
|  |  |  |  |  |  |  | **1kGP_EUR** | **Serbian** |  |  |
| PSPH | rs79451216 | chr7:g.56019730G>A | MODERATE | missense | 36% | 30% | 0.001 | 0.163 | 163 | **1.9E-12** |
| PCDHB4 | rs147934595 | chr5:g.141123572C>A | MODERATE | missense | 43% | 46% | 0.003 | 0.222 | 74 | / |
|  | rs144689137 | chr5:g.141123435G>A | LOW | synonymous | 38% | 45% | 0.008 | 0.208 | 26 | **2.0E-39** |
| MRC1 | rs71497224 | chr10:g.17849727C>T | LOW | synonymous | 93% | 94% | 0.0109 | 0.771 | 70.73 | 5.1E-02 |
|  | rs374113136 | chr10:g.17833763G>A | LOW | synonymous | 67% | 61% | 0.0497 | 0.41 | 8.25 | 3.9E-03 |
| KIR2DL1 | rs79002558 | chr19:g.54773524A>G | MODERATE | missense | 15% | 11% | 0.002 | 0.066 | 33 | **1.0E-19** |
|  | 19:54775225 | chr19:g.54775225A>G | MODERATE | missense | 21% | 13% | 0.004 | 0.085 | 21.25 | **1.0E-03** |
|  | 19:54775226 | chr19:g.54775226T>C | LOW | synonymous | 21% | 13% | 0.004 | 0.085 | 21.25 | **9.8E-04** |
| KIR3DL1 | rs149760111 | chr19:g.54821568C>T | MODERATE | missense | 36% | 36% | 0.0149 | 0.198 | 13.29 | 6.1E-02 |
| KIR2DL3 | rs149224427 | chr19:g.54742075C>G | MODERATE | missense | 54% | 52% | 0.0318 | 0.356 | 11.19 | 1.5E-02 |
| HLA-DRB5 | rs147439581 | chr6:g.32518589C>T | MODERATE | missense | 57% | 65% | 0.0398 | 0.433 | 10.88 | / |
|  | rs41559420 | chr6:g.32519465G>A | MODERATE | missense | 20% | 22% | 0.0239 | 0.122 | 5.10 | **5.2E-17** |
| CDSN | rs145583110 | chr6:g.31116168C>T | MODERATE | missense | 10% | 13% | 0.006 | 0.059 | 9.83 | / |
| RHPN2 | rs147870656 | chr19:g.32999658G>T | MODERATE | missense | 13% | 11% | 0.007 | 0.059 | 8.43 | **5.0E-19** |
| BTNL2 | rs28362679 | chr6:g.32396116G>A | MODERATE | missense | 23% | 22% | 0.0159 | 0.115 | 7.23 | **5.4E-21** |
| DND1 | 5:140671341 | chr5:g.140671341G>A | LOW | synonymous | 16% | 14% | 0.0109 | 0.077 | 7.06 | / |
| HTR3A | rs33940208 | chr11:g.113975355C>T | LOW | synonymous | 7% | 13% | 0.008 | 0.052 | 6.5 | **5.8E-07** |
| GAL3ST3 | rs147282818 | chr11:g.66043629C>T | LOW | synonymous | 21% | 13% | 0.0139 | 0.09 | 6.47 | / |
| TNXB | rs17207895 | chr6:g.32052735T>C | MODERATE | missense | 11% | 25% | 0.0189 | 0.101 | 5.34 | **3.3E-31** |
| HLA-DQB1 | rs41552812 | chr6:g.32664912C>T | MODERATE | missense | 21% | 23% | 0.0239 | 0.125 | 5.23 | **1.2E-06** |

**Supplementary Table S3** Variants detected as frequent (MAF≥5) in the Serbian population compared to the European population of 1kGP and their sex representation. The one-tailed Fisher’s exact test was used to measure significant differences in allele frequencies for the variants that had a fold increase >5 in comparison with the European population from 1kGP. After the Bonferroni correction the P-value threshold was P<0.002. The bolded values are considered statistically significant and rounded to two significant figures.

| **Gene** | **Variant** | **AAS** | **MutPred 2 score** | **MutPred2 - Affected PROSITE and ELM**  **Motifs** | **MutPred2 - Molecular mechanisms** |
| --- | --- | --- | --- | --- | --- |
| PSPH | rs79451216 | R49 W | 0.637 | ELME000063 - CK1  Phosphorylation site  ELME000102 -  NRD cleavage site  ELME000108 -  PCSK cleavage site  PS00005 - Protein kinase C  phosphorylation site | Loss of Relative solvent accessibility  Loss of ADP- ribosylation at R49  Altered Metal binding |
|  |  | R49G | 0.725 |  | Loss of Relative solvent accessibility  Loss of Helix  Gain of ADP- ribosylation at R50  Altered Metal binding, Gain of  Methylation at R50 |
| KIR2 DL1 | rs79002558 | S88R | 0.573 | ELME000008 - PKA  Phosphorylation site  ELME000012 - di Arginine retention/ retrieving signal  ELME000062 - PKA  Phosphorylation site  ELME000063 - CK1  Phosphorylation site  ELME000070 - N-  glycosylation site  ELME000102 -  NRD cleavage site  ELME000108 -  PCSK cleavage site | Loss of Relative solvent accessibility  Altered Trans- membrane protein  Altered Ordered interface  Gain of Allosteric site at R89  Gain of ADP- ribosylation at S88  Altered DNA binding  Altered Metal binding  Loss of N-linked glycosylation at N84 |
| BTNL 2 | rs28362679 | S334 L | 0.514 | ELME000053 - GSK3  phosphorylation site  ELME000062 - PKA  Phosphorylation site  ELME000239 -  USP7 binding motif  PS00007 - Tyrosine kinase phospho-  rylation site 1 | Altered Transmembrane protein  Loss of B-factor |
|  |  | S334 W | 0.691 |  |  |
| HLA- DQB1 | rs41552812 | D89N | 0.503 |  | Altered Ordered  Gain of Relative solvent accessibility |

**Supplementary Table S4** Variants detected as frequent in Serbian population that MutPred2

[8] predicted as affecting protein function with MutPred2 score and Affected PROSITE and ELM Motifs**.**

| **Gene** | **GO-BPO annotations** |
| --- | --- |
| **PSPH** | L-serine metabolic process |
| **PCDHB4** | chemical synaptic transmission  nervous system development  synapse assembly |
| **KIR2DL1** | natural killer cell inhibitory signalling pathway  regulation of immune response  immune response |
| **HTR3A** | chemical synaptic transmission |
| **HLA-DRB5** | antigen processing and presentation of exogenous peptide antigen via MHC class II  interferon-gamma-mediated signalling pathway  T cell receptor signalling pathway |
| **HLA-DQB1** | Immunoglobulin production involved in immunoglobulin mediated immune response  humoral immune response mediated by circulating immunoglobuli  antigen processing and presentation of exogenous peptide antigen via MHC class II  T cell signalling pathway  interferon-gamma-mediated signalling pathway |
| **TNXB** | collagen metabolic process  elastic fiber assembly |

**Supplementary Table S5** GO-BPO annotations for genes with variants overrepresented and significant in the Serbian population sample. GO terms common for two or more genes are colored; red – immune response, blue – regulation of immune response, green – chemical synaptic transmission.

**SUPPLEMENTARY REFERENCES**

[1.] Li, H. *et al.* The Sequence Alignment/Map format and SAMtools.

*Bioinformatics* **25**, 2078–2079 (2009).

[2.] Ng, P. C. *et al.* Genetic variation in an individual human exome. *PLoS Genet.*

**4**, e1000160 (2008).

[3.] Challis, D. *et al.* The distribution and mutagenesis of short coding INDELs from 1,128 whole exomes. *BMC Genomics* **16**, 143 (2015).

[4.] McLaren, W. *et al.* The Ensembl Variant Effect Predictor. *Genome Biol.* **17**, 122 (2016).

[5.] Kumar, P., Henikoff, S. & Ng, P. C. Predicting the effects of coding non- synonymous variants on protein function using the SIFT algorithm. *Nat Protoc* **4**, 1073–1081 (2009).

[6.] Adzhubei, I. A. *et al.* A method and server for predicting damaging missense mutations. *Nat. Methods* **7**, 248–249 (2010).

[7.] Alexander, D. H., Novembre, J. & Lange, K. Fast model-based estimation of ancestry in unrelated individuals. *Genome Research* **19**, 1655–1664 (2009).

[8.] Pejaver, V. *et al.* MutPred2: inferring the molecular and phenotypic impact of amino acid variants. *bioRxiv* 134981 (2017) doi:10.1101/134981.
